# Dosimetrically triggered adaptive radiotherapy for head and neck cancer: Considerations for the implementation of clinical protocols

**DOI:** 10.1002/acm2.14095

**Published:** 2023-07-13

**Authors:** Ana M. Barragán‐Montero, Geneviève Van Ooteghem, Damien Dumont, Sara Teruel Rivas, Edmond Sterpin, Xavier Geets

**Affiliations:** ^1^ UCLouvain Center of Molecular Imaging Radiotherapy and Oncology (MIRO) Brussels Belgium; ^2^ Department of Radiation Oncology Cliniques universitaires Saint‐Luc Brussels Belgium; ^3^ Department of Oncology Laboratory of Experimental Radiotherapy KU Leuven Leuven Belgium

**Keywords:** adaptive radiotherapy, dosimetrically triggered adaptation, external beam radiotherapy‐photons, head and neck cancer, protocol implementation

## Abstract

**Purpose:**

Defining dosimetric rules to automatically detect patients requiring adaptive radiotherapy (ART) is not straightforward, and most centres perform ad‐hoc ART with no specific protocol. This study aims to propose and analyse different steps to design a protocol for dosimetrically triggered ART of head and neck (H&N) cancer. As a proof‐of‐concept, the designed protocol was applied to patients treated in TomoTherapy units, using their available software for daily MVCT image and dose accumulation.

**Methods:**

An initial protocol was designed by a multidisciplinary team, with a set of flagging criteria based only on dose‐volume metrics, including two action levels: (1) surveillance (orange flag), and (2) immediate verification (red flag). This protocol was adapted to the clinical needs following an iterative process. First, the protocol was applied to 38 H&N patients with daily imaging. Automatic software generated the daily contours, recomputed the daily dose and flagged the dosimetric differences with respect to the planning dose. Second, these results were compared, by a sensitivity/specificity test, to the answers of a physician. Third, the physician, supported by the multidisciplinary team, performed a self‐analysis of the provided answers and translated them into mathematical rules in order to upgrade the protocol.

The upgraded protocol was applied to different definitions of the target volume (i.e. deformed CTV + 0, 2 and 4 mm), in order to quantify how the number of flags decreases when reducing the CTV‐to‐PTV margin.

**Results:**

The sensitivity of the initial protocol was very low, specifically for the orange flags. The best values were 0.84 for red and 0.15 for orange flags. After the review and upgrade process, the sensitivity of the upgraded protocol increased to 0.96 for red and 0.84 for orange flags.

The number of patients flagged per week with the final (upgraded) protocol decreased in median by 26% and 18% for red and orange flags, respectively, when reducing the CTV‐to‐PTV margin from 4 to 2 mm. This resulted in only one patient flagged at the last fraction for both red and orange flags.

**Conclusion:**

Our results demonstrate the value of iterative protocol design with retrospective data, and shows the feasibility of automatically‐triggered ART using simple dosimetric rules to mimic the physician's decisions. Using a proper target volume definition is important and influences the flagging rate, particularly when decreasing the CTV‐to‐PTV margin.

## 1 INTRODUCTION

During radiotherapy treatments, some patients may experience important day‐to‐day or inter‐fractional anatomical modifications in the tumour region (cavity filling, weight loss, tumour shrinkage, …). It is well‐known that these changes can significantly affect the actual dose distribution, no longer mirroring the treatment plan elaborated from the pre‐treatment planning image. Adaptive radiotherapy (ART)^1^, ^2^ overcomes these inter‐fractional anatomical modifications by adapting the treatment plan according to the daily anatomy of the patient. Thus, ART aims to ensure optimal target coverage and healthy tissue sparing along the treatment, which may consequently improve treatment quality.^3^, ^4^, ^5^, ^6^

However, ART is a very resource‐intensive task, since it requires the acquisition of volumetric images, the generation and validation of new target and organ contours, and the optimization and approval of a new plan. Despite the evolution of the treatment technologies (better in‐room imaging, auto‐segmentation tools, faster plan optimization software, etc), the technical and logistic difficulties are still today a bottleneck for the broad clinical implementation of ART for every patient and treatment fraction.^7^ Moreover, the clinical added value of a daily adaptation strategy is not well estimated yet for several localisations, including head and neck cancer patients (H&N). For instance, there is considerable literature investigating the need of replanning for H&N, which reported moderate to low replanning rates ranging from 20%−30% to less than 10%−5% of the total number of patients analysed.^8^, ^9^, ^10^, ^11^ Therefore, developing tools to automatically select the patients who would really benefit from ART and to decide the optimal frequency of replanning is one of the keys towards a successful clinical implementation of ART.

In order to design automatic patient selection tools for ART, multiple approaches can be used. The simplest approaches rely exclusively upon volumetric and density changes seen in the new image (daily CBCT/MVCT, repeated CT, or MR) to trigger adaptation.^10^, ^11^ However, it has been reported that important volumetric changes often had an inconspicuous dosimetric impact for high energy X‐rays treatments.^12^ Therefore, a proper analysis of the dosimetric differences between the new and the planning image is recommended in order to determine whether ART should be applied or not. In the last few years, several companies have launched commercial software that automatically perform the dose calculation in the new image and generate new contours. The new contours are obtained either by propagating the planning volumes using deformable image registration,^13^, ^14^, ^15^, ^16^, ^17^ by using automatic (e.g. atlas‐based or machine learning) segmentation models on the new image,^18^ or by a combination of both.^19^ The dose on the new anatomy and the new contours are then used to generate dose volume histograms (DVH) and related dose metrics for the current fraction, as well as to estimate the evolution of these metrics at the end of the treatment through dose accumulation techniques.^20^, ^21^, ^22^ In addition, some of these software enable the user to define different thresholds or tolerances on the DVHs metrics, often called *flagging* criteria, to automatically identify the patients who are susceptible to benefit from replanning.

However, despite the large availability of commercial decision support software for ART, the users still know very little on how best define these dosimetric flagging criteria in a clinically‐relevant way, and the consequences on the flagging rate when using more or less conservative values. The “Patterns of Practice for adaptive and real‐time radiation therapy (POP‐ART RT)”[7] from 2020, which analyses the practice of 177 centres all over the world, reported that although 55% of the participants did perform ART for H&N cancer, only 10% used any specific online or offline protocols (with pre‐defined flagging criteria or action levels). The remaining 45% performed ad‐hoc ART with no specific protocol, which reflects a lack of consistency and standardisation in the clinical practice of ART.

Recently, ETHOS technology (Varian Medical Systems)^19^ revolutionised the landscape of adaptive therapy, with a completely automated workflow based on artificial intelligence and deformable registration. If their adaptive workflow, the system generates new contours on the daily CBCT and proposes an adapted plan for every single fraction, regardless the magnitude of the dose deviations with respect to the planned treatment. This requires both a physician and a physicist to be in the console at any time, the former in charge of reviewing (and eventually correcting) the new contours and evaluating the new dose distribution, and the latter to approve the new treatment plan. In cases where the deviations are low, the waiting time to get the adapted plan could be economised by using smart flagging criteria that will advise us to directly use the initially planned treatment. This can be especially relevant in cases where the complexity is high and the optimization time is longer (e.g. head and neck VMAT cases) or when there is a shortage in caregivers. Note that, in the current version of ETHOS, the user can only choose at the planning stage to use an adaptive or non‐adaptive workflow, with no possibility to change that throughout the treatment.

The present study aims to capture and analyse the different steps in the design of a clinical protocol for dosimetrically triggered treatment adaptation of H&N cancer, and extract potential recommendations to increase the consistency and reproducibility of clinical practice for ART. The protocol was designed by a multidisciplinary group of physicians and physicists, and initially included two action levels: (1) surveillance, marked with an orange flag, and (2) immediate online verification request, marked with a red flag. In addition, it compared several tolerances for each action level in order to address the influence of more or less conservative action levels in the rate of adaptation. The protocol was applied to a retrospective cohort of 38 patients treated in a Tomotherapy machine with daily MVCT imaging and available software for dose accumulation. The results of the automatic protocol were compared to the answers of a physician, who manually went through all patients indicating the action level needed for each fraction and organ. The protocol was then upgraded, following an iterative process, to ensure that the two considered action levels accurately matched the clinical needs, in this case represented by the answers of the physician. To finish, we applied this final (upgraded) protocol to different definitions of target volumes (i.e. deformed CTV + 0, 2, and 4 mm) to evaluate how the choice of volume influences the flagging rate.

## 2 MATERIAL AND METHODS

### 2.1 Automatic decision support software for ART

**FIGURE 1:**
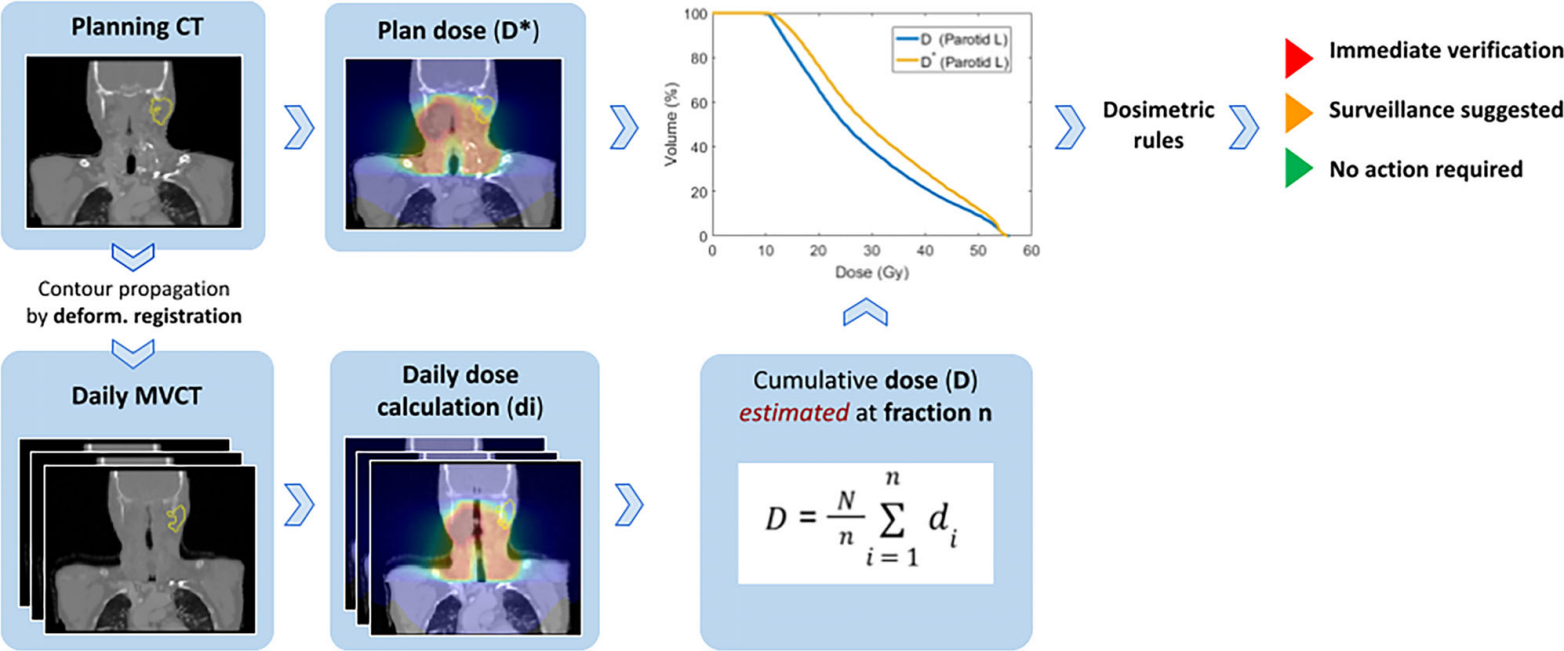
Illustration of the automatic decision support tool used to flag the candidate patients for ART.

The automatic platform for adaptive therapy developed by *21^st^ Century Oncology* (Madison, WI)[16] was used to monitor the delivered dose using daily setup Megavoltage CT images (MVCT) acquired from the Tomotherapy units installed in the Cliniques Universitaires Saint‐Luc, Brussels, Belgium. Figure 1 illustrates the process to automatically flag the candidate patients for ART. For each fraction, a merged image is generated by rigidly registering the MVCT to the planning CT, using the setup information from the Tomotherapy archive, and filling the missing information in the MVCT with the planning CT in both radially and superior/inferior directions.^23^ The dose is recomputed directly on this merged MVCT image with their in‐house convolution‐superposition dose engine,^24^ using a user‐specific MVCT calibration curve. In the next step, a Morphons algorithm^25^ is used to calculate the deformation vector field (DVF) between the planning CT and the merged MVCT. This DVF is used to propagate the contours from the planning CT to the daily MVCT, as well as to warp the recomputed dose to the planning CT for dose accumulation. The platform then displays the accumulated and daily doses, as well as the corresponding DVHs and metrics of interest, such as the dose to 95% of the volume (D95) or the volume covered by the 20 Gy isodose line (V20), among others. All this information is presented in *html* format and can be saved for data post‐processing.

An in‐house Matlab set of functions was created to read the *html* information and analyse the data according to different flagging criteria detailed in Section 2.2. At each fraction *n*, the final delivered dose (***D***) was estimated by accumulating the daily delivered dose till fraction n, and adding up the remaining fractions by assuming that they would deliver a dose equal to the average of the fractions delivered so far, that is

$$\begin{array}{ccc} & & D\left(\mathrm{esti}\mathrm{mated}\;\mathrm{at}\;\mathrm{frac}\mathrm{tion}\;n\right)\;\\ & & \;=\sum_{i\;=\;1}^{n}d_{i}+\frac{\sum_{i\;=\;1}^{n}d_{i}}{n}\cdot \left(N-n\right)=\frac{N}{n}\cdot \sum_{i\;=\;1}^{n}d_{i} \end{array}$$

where ***N*** is the total number of fractions, ***n*** is the number of fractions delivered so far, and ***d_i_*** is the dose at the (already delivered) *i*‐th fraction. Note that the dose accumulation is done by using the same DVF previously computed to propagate the contours. The flagging criteria (Section 2.2) for the considered region of interests (ROIs) were based on specific thresholds for the difference between relevant DVH metrics computed on the estimated ***D*** at each fraction and the planned dose (D*). The specific thresholds were established based on clinical judgement.

### 2.2 Initial protocol design and flagging criteria

After a multidisciplinary meeting including radiation oncologists and physicists, a set of flagging criteria was established, based on specific thresholds (**t_j_**) on relevant DVH metrics (**m_j_**) for the target volumes and organs at risk, following recommended values in the literature.^26^, ^27^, ^28^ The threshold values for each metric *j* (***t_j_***) are presented in Table 1. For the target volumes, only dose coverage metrics (D99 and D95) were evaluated, and the threshold **t** was computed relative to the dose prescription (D_pre_) for each target. For instance, 95% of the PTV volume should receive at least 95% of the prescribed dose, that is, D_95_(PTV) > 95%D_pre_. Each type of target was evaluated separately, using three categories: PTV_T (primary tumour), PTV_NL (left node), and PTV_NR (right node). For the organs at risk, the threshold **t** was a reference value specific to each organ, for example for the spinal cord, D_2_ < 48 Gy for H&N.

**TABLE 1:** The first column contains the index ***j*** of the “ROI (region of interest)—DVH metric” (***m_j_***) combinations presented in the second column, while the third column displays the value of the corresponding thresholds ***t_j_***.

*j*	ROI—DVH metric (m_j_)	*t_j_*
1	PTV—D_99_	90% Dpre
2	PTV—D_95_	95% Dpre
3	Spinal cord—D_2_	48 Gy
4	Brainstem—D_2_	60 Gy
5	Parotid ipsi.—D_mean_	30 Gy
6	Parotid cont.—D_mean_	26 Gy
7	Larynx—D_5_	45 Gy
8	Oral cavity—D_mean_	30 Gy
9	Mandible—D_2_	70 Gy
10	Superior constrictors muscles—D_mean_	45 Gy

Based on these thresholds, two different flagging approaches were implemented: (a) surveillance suggested (*orange flag*), when the difference between the estimated ***m_j_*** (Section 2.1) and planning m_j_* was above a specific tolerance (***tol***, expressed as a percentage %) of ***t_j_***, or below in case of the target coverage; and (b) immediate verification required (*red flag*), when the difference between estimated ***m_j_*** and planned m_j_* was above the percentage tolerance (***tol***, %) of m_j_* AND the estimated ***m_j_*** was above ***t_j_*,** or below in case of target coverage related metrics. If neither (a) nor (b) was applicable, the patient was flagged green and no action was required. Those criteria were meant to avoid futile flags, in cases where the initial m_j_* could already not meet the tolerance ***t_j_***, but was knowingly accepted. In practice, “surveillance suggested” meant that the radiation oncologist in charge of the treatment should be notified the same day about the changes offline (i.e. can be done after the treatment), in order to keep a closer eye for the next fractions, but the treatment for the current fraction can be delivered. In contrast, “immediate verification” should require an urgent “online” notification to the medical doctor, who should verify the estimated dose online (i.e. the patient is on the treatment couch) and decide if the fraction can be delivered as it is or if it requires an immediate adapted plan.

Three different tolerance levels were used to simulate increasing conservativeness in the flagging criteria, that is ***tol*** = 10% for Level 1, ***tol*** = 5% for Level 2, and ***tol*** = 2.5% for Level 3. In order to clarify the implementation of orange and red flags, they are expressed as mathematical expressions in Table 2 (using the same notation above) and some examples are also given.

**TABLE 2:** Implementation of orange and red flags and examples for specific cases. ***m_j_*** stands for the ***j*** DVH metric (see Table 1) estimated at each fraction (Section 2.1), whereas m_j_* refers to the planned ***j*** DVH metric. The tolerance value for a given level is represented by ***tol***, and ***t_j_*** corresponds to the threshold value for a given DVH metric, which is indicated in Table 1.

Flag type	Orange	Red
Flag conditions when m_j_* satisfies t_j_	m_j_ − m_j_* **>** ***tol × t_j_*** *(OAR overdose)* m_j_ − m_j_* **<** −***tol × t_j_*** *(Target undercoverage)*	1) m_j_ − m_j_* **>** tol × m_j_* *and* ***2) m_j_ > t_j_*** *(OAR overdose)* 1) m_j_ − m* **<** −tol × m_j_* *and* ***2) m_j_ < t_j_*** *(Target undercoverage)*
Flag conditions when m_j_* violates t_j_	***–***	m_j_ − m_j_* **>** tol × m_j_* *(OAR and target overdose)* m_j_ − m* **<** −tol × m_j_* *(Target undercoverage)*
*Example 1* ***j*** = 2 ***m_2_ =*** PTV − D95 ***D_pre_*** = 70 Gy ***t_1_*** = 95% of ***D_pre_*** = 66.5 Gy ***tol*** = 10% (Level 1)	D95 – D95* < −6.65 Gy	D95 – D95* < −10% of D95* AND D95 < 66.5 Gy
*Example 2* ***j*** = 5 ***m_5_*** = Parotid ipsi − D_mean_ ***t_5_*** = 30 Gy ***tol*** = 10% (Level 1)	D_mean_ − D_mean_* > 3 Gy	D_mean_ − D_mean_* > 10% of D_mean_* AND D_mean_ > 30 Gy

For cases in which the planned m_j_* already violates the clinical threshold ***t_j_***, for example D_mean_* > 30 Gy for the ipsilateral parotid, a red flag was triggered when the difference between estimated ***m_j_*** and planned m_j_* was above (or below for target coverage) the percentage tolerance (***tol***, %) of m_j_*, that is, using only the first condition of the *red flag*.

Note that the implemented protocol evaluated each fraction independently, that is the protocol was blind to the number of flags in the previous fractions and the fraction number. Differences below 0.1 Gy in every flag condition were not considered.

### 2.3 Patient database

The protocol was applied to a retrospective patient database that included 38 H&N cancer patients treated with Tomotherapy units. Daily MVCT images were acquired for setup positioning and then used for dose monitoring as explained in Section 2.1. The patient database included fractionation schedules that ranged from 30 to 35 sessions, and target dose prescription with values in the range of 52.5–70 Gy.

Visual inspection of all propagated contours from planning to daily images through deformable registration (Section 2.1) was performed by an expert physician for each patient at each fraction in the database, to ensure that the daily contours were generated correctly.

### 2.4 Protocol review and upgrade

**FIGURE 2:**
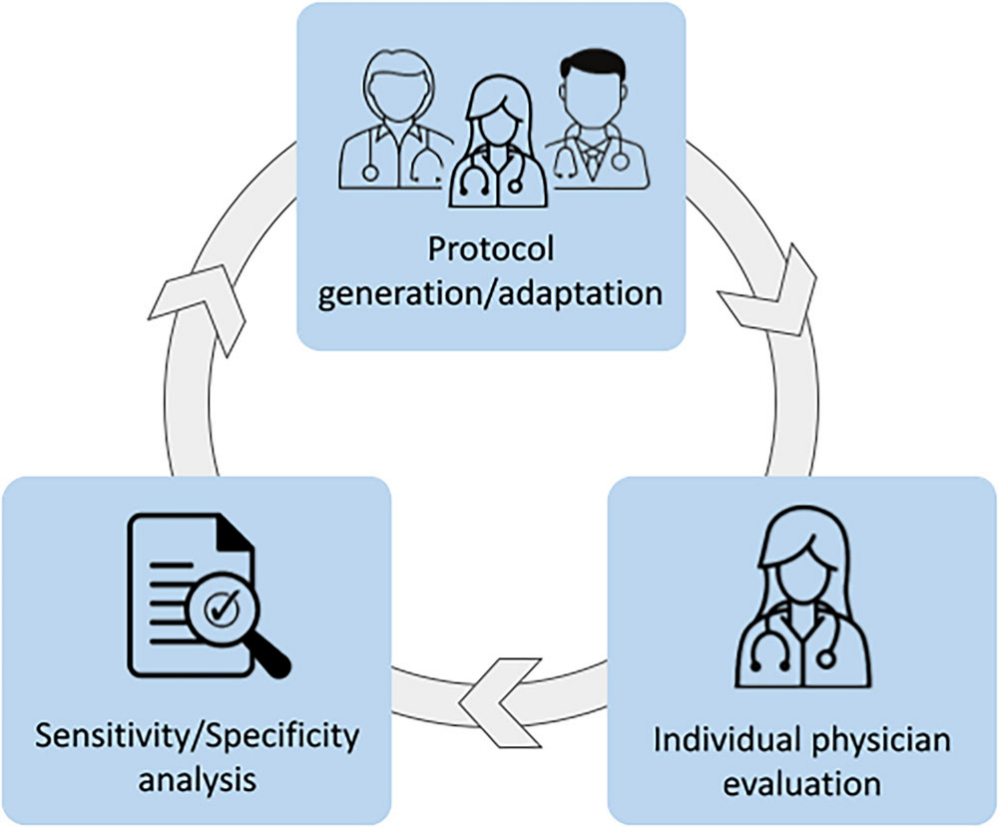
Three‐step loop process to evaluate and adapt the initially generated (theoretical) flagging protocol.

In order to benchmark and eventually adapt and/or improve the initial flagging protocol described in Section 2.2, we followed a three‐step loop process. First, an expert physician independently evaluated all the DVHs for the considered volumes for every patient and marked every fraction with a green (no action required), orange (surveillance suggested) or red flag (immediate verification required), accordingly, based on her clinical experience. In order to be consistent with the implemented protocol, the physician looked at each fraction as independent data, without taking into account the number of flags in the previous fractions nor the fraction number (i.e. beginning, middle or end of the treatment). Note that this physician had only access to DVHs data, and not to the associated three‐dimensional dose distribution. Second, a sensitivity/specificity analysis was performed to compare the results obtained from the initial flagging protocol (including the different tolerances) with the physician's answers. Third, the physician, together with the help of the multidisciplinary team (other physicians and physicists), performed a self‐analysis of the provided answers and tried to translate them into systematic mathematical rules in order to upgrade the protocol. Note that we assume that the ground truth for the actual clinical needs regarding the action levels (red/orange) is represented by the answers of the physician. If needed, these steps were repeated until the multidisciplinary team estimated that the protocol was optimal (i.e. reproducing accurately the clinical decisions to trigger a red/orange flag). Figure 2 illustrates the process for protocol review and optimization.

### 2.5 Evaluation of the flagging rate for different definitions of the target volume

The initial protocol (Section 2.2) and the upgraded protocol during the review process (Section 2.4) were both evaluated on a daily PTV, obtained after deformable registration from the planning image (Section 2.1). Although deforming a PTV is questionable, we still chose to use this volume for simplicity in our workflow, since this was the daily target volume provided by the software and displayed in the graphical interface used by the physician. It is important to note that, in order to design a protocol, the target volume definition becomes irrelevant as long as the physician applies the same rules for all definitions (which was confirmed by the physician). Nevertheless, it is pertinent to compare the effect of different target volume definitions on the flagging rate, which was done for the final (upgraded protocol). First, the flagging rate using the final (upgraded) protocol on the daily deformed PTV was compared to that on the deformed CTV with a margin extension of 4 mm (i.e. the planning CTV‐to‐PTV margin). Second, the final (upgraded) protocol was applied to the deformed CTV with 2 mm margin extension, and the deformed CTV without any margin extension (+0 mm). This served to illustrate how the flagging rate changes with reduced margins, simulating a daily adaptation context, where the contribution from the systematic setup error can be removed from the margin.

## 3 RESULTS

### 3.1 Initial flagging protocol *(flagging rules from* Table 2)

**FIGURE 3:**
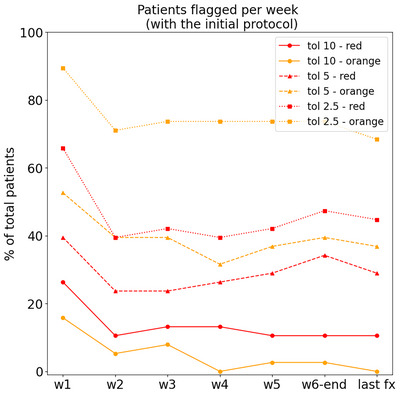
Percentage of total patients with at least one flag (orange or red) per week (w1 to w6) and for the last fraction of the treatment (last fx), for the flagging criteria included in the initial protocol, evaluating the three considered action levels with different tolerance levels.

**FIGURE 4:**
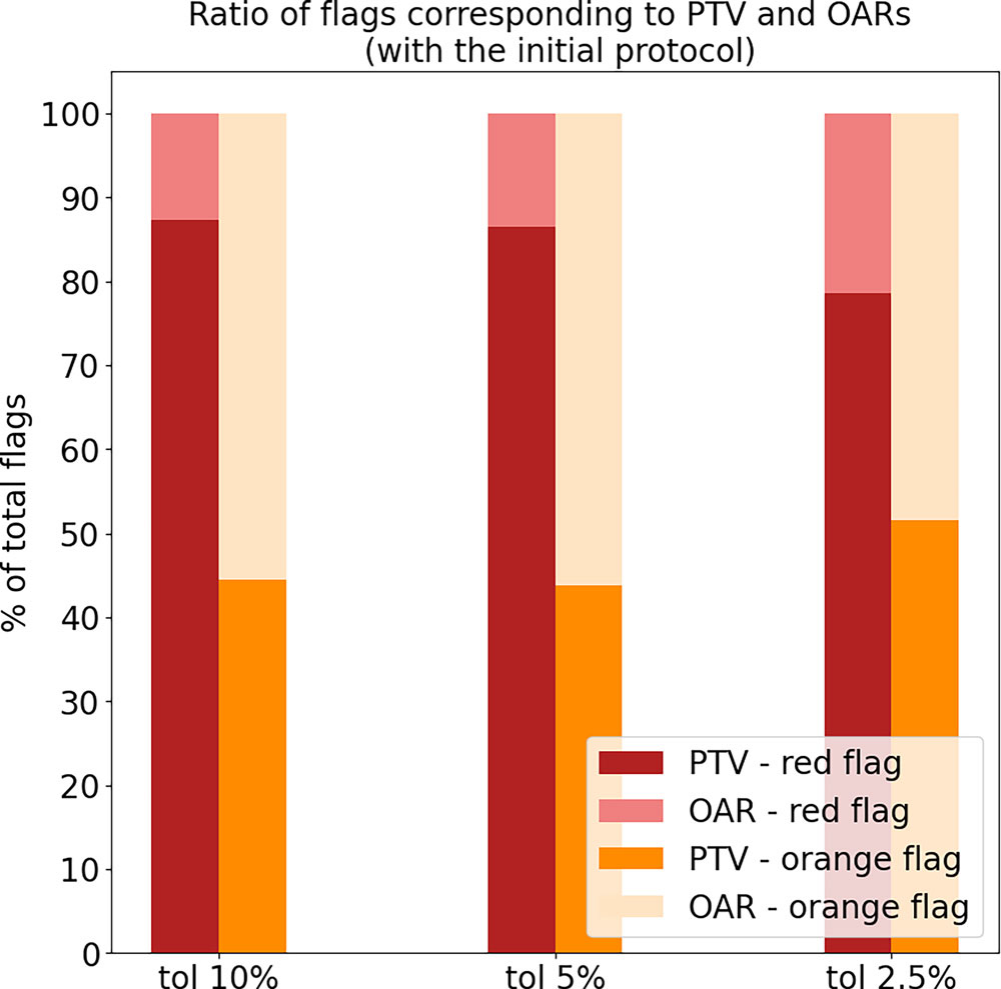
Ratio of total orange and red flags (counting all fractions and all patients) corresponding to PTV and OARs respectively, for the three considered tolerance levels of the initial protocol.

In order to analyse the weekly evolution of the flagging rate, the number of patients with at least one flag (orange/red) is reported per week (set of five fractions) in Figure 3. Note that the results presented in this section are simply obtained by applying the criteria in Table 2. As expected, the number of flagged patients increased with decreasing tolerance, reaching up to an average of 75% of the total patients flagged per week with orange and 46% with red, for the most conservative action level (**tol** = 2.5%). Figure 3 shows also that the flagging criteria evaluated on the first week (fractions 1–5) clearly overestimated the actual deviations at the end of the treatment (i.e., patients that were still flagged at the last fraction), with a difference of 10%–20% in the total number of flagged patients (w1 vs. last‐fx in Figure 3). From week 2 onwards, the number of flagged patients per week remained more stable for all action levels, being closer to the results for the last fraction (maximum absolute difference of 8%), and therefore, being a better indication of the actual final dose deviations. Figure 4 shows the percentage of total flags (counting all fractions and all patients) assigned to the PTVs and the OARs, respectively. For the red condition, more than 75% of total flags corresponded to the PTV, while the orange condition flagged PTVs and OARs almost at the same rate.

### 3.2 Protocol review

**FIGURE 5:**
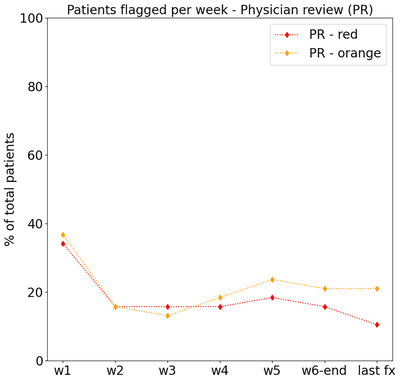
Percentage of total patients with at least one flag (orange or red) per week (w1–w6) and for the last fraction of the treatment (last fx), for the flagging criteria applied by the physician.

One physician from the team was designated to perform the evaluation of the DVHs for all considered volumes, fractions and patients. The number of patients flagged per week by the physician is presented in Figure 5. For the first week (w1) the percentage of patients with at least one flag reached almost 40%, and it decreased to 20% or less for the rest of the fractions. The flagging rate applied by the physician was in between the first (tol = 10%) and second (tol = 5%) tolerance levels of the initial protocol. Note that directly comparing the flagging rates from the initial protocol and the one applied by the physician does not make sense, since one can have the same flagging rate without necessarily matching the individual flags, as it is shown by the low sensitivity results (Table 3). The flagging rates are provided instead to illustrate the amount of patients flagged by each approach.

**TABLE 3:** Sensitivity and specificity analysis for the results obtained with the initial protocol (for the three considered tolerance levels) in comparison to the answers from the physician (assumed as ground truth).

	RED			ORANGE		
	tol = 10%	tol = 5%	tol = 2.5%	tol = 10%	tol = 5%	tol = 2.5%
**True positive (TP)**	125	182	269	2	3	45
**False positive (FP)**	93	273	490	34	477	1447
**False negative (FN)**	194	137	50	263	263	263
**True negative (TN)**	11281	11101	10884	11394	10950	9938
**Sensitivity TP/(TP + FN)**	0.39	0.57	0.84	0.01	0.01	0.15
**Specificity TN/(TN + FP)**	0.99	0.98	0.96	1.0	0.96	0.87

Regarding the type of volume flagged (PTV vs. OAR), the answers from the physician were very different from the results obtained by the initial protocol: 100% of the red flags (counting all patients and fractions) were on the PTV, and similarly for the orange flags, where almost all flags were on the PTV (99.62%) except a few flags on the ipsilateral parotid (0.38%).

A sensitivity/specificity analysis was performed to compare the flags from the initial protocol to those applied by the physician (Table 3). Note that the number of True Negatives (TN) is very high, due to the fact that most of the organs and fractions did not have any flag. This results in a very high specificity, which cannot serve to draw any conclusions. Therefore, we focus on the values of the sensitivity instead, which is a better measurement of the accuracy of the protocol.

The highest sensitivity for the red flags (0.84) was obtained for the level with tolerance 2.5%. However, it was at the expense of a very high number of False Positives (FP = 490). For the orange flags, the sensitivity was extremely low (below 0.15 for all tolerance levels). These results demonstrate that the initially designed protocol did not reproduce at all the clinical decisions, represented by the answers from the physician.

### 3.3 Protocol upgrade *(flagging rules from* *Table* *4*)

The initially designed protocol (Table 2) was upgraded to include new rules elaborated after a self‐analysis of the physician during the review process (Table 4), that is the physician analysed her own answers and tried to translate them into mathematical rules with the help of the physicists’ team. In particular, the physician reported that the initial protocol was too simplistic and that more sophisticated criteria were needed. For instance, the flagging criteria for the PTVs was upgraded so that a flag was not triggered anymore based on the violation of a single metric but rather on a combination of two metrics (***m_1_*** and ***m_2_*** from Table 1). Regarding the flagging criteria for the OARs, the physician indicated that distinguishing between PTV volumes, serial, and parallel organs was mandatory, and therefore, different flagging criteria were upgraded accordingly for each type of volume. The upgraded protocol is summarised in Table 4. A tolerance level was only applied to the OARs flagging criteria and was fixed to 20%.

**TABLE 4:** Upgraded protocol. All metrics (***m_j_***) and thresholds (***t_j_***) remained the same as for the initial protocol (see Table 1), only new combinations and rules were applied. ***m_j_*** stands for the ***j*** DVH metric (see Table 1) estimated at each fraction (Section 2.1), whereas m_j_* refers to the planned ***j*** DVH metric. The tolerance value is represented by ***tol*** and was fixed to 20%, and ***t_j_*** corresponds to the threshold value for a given DVH metric, which is indicated in Table 1.

Flag type	Orange	Red
Flag conditions *PTV*	m_1_ < m_1_* AND **m_1_ < t_1_** AND **m_2_ ≥ t_2_** OR m_2_ < m_2_* AND **m_2_ < t_2_** AND **m_1_ ≥ t_1_**	m_1_ < m_1_* AND m_2_ < m_2_* AND **m_1_ < t_1_** AND **m_2_ < t_2_**
Flag conditions *serial OAR*	m_j_ − m_j_* > *tol × t_j_* AND ***m_j_* < t_j_**	*m_j_* > m_j_* AND ***m_j_* > t_j_**
Flag conditions *parallel OAR*	m_j_ − m_j_* > *tol × t_j_* AND ***m_j_* > t_j_**	***–***

A new sensitivity/specificity analysis was performed between the answers of the physician and the new upgraded protocol (Table 5). The sensitivity increased to 0.96 for the red flags and to 0.84 for the orange flags, demonstrating a much better match between the upgraded protocol and the answer from the physician, in comparison to the initial protocol. In particular, the number of True Positives (TP) increased to 307 for red flags and 221 for orange flags. Although there were still some apparent False Negatives (FN = 12 red and FN = 42 orange), the upgraded protocol was not totally blind to these cases, since all 12 FN for the red flags were actually FP for the orange flags, and 41 out the 42 FN for the orange flags were counted as FP for the red flags. This leads to only one case missed by the upgraded protocol. This can also be observed with the global sensitivity/specificity test (Table 5, Column 3), where we compared the organs and fractions flagged, regardless of the colour of the flag. Indeed, the global sensitivity was equal to 1 (with only one FN) and the specificity was equal to 0.97.

**TABLE 5:** Sensitivity and specificity analysis for the results obtained with the upgraded protocol in comparison to the answers from the physician (assumed as ground truth). *All 12 FN for the red flags were counted as FP for the orange flags, and 41 out of the 42 FN for the orange flags were counted as FP for the red flags. As for the FP, those 333 global flags concerned seven patients in total. The third column represents the “global” results, where we compared the organs and fractions flagged by the upgraded protocol and the physician, regardless of the colour of the flag.

	RED	ORANGE	GLOBAL
**True positive (TP)**	307	221	581
**False positive (FP)**	112	274	333
**False negative (FN)**	12*	42*	1
**True negative (TN)**	11262	11156	10778
**Sensitivity TP/(TP + FN)**	0.96	0.84	1.0
**Specificity TN/(TN + FP)**	0.99	0.98	0.97

**FIGURE 6:**
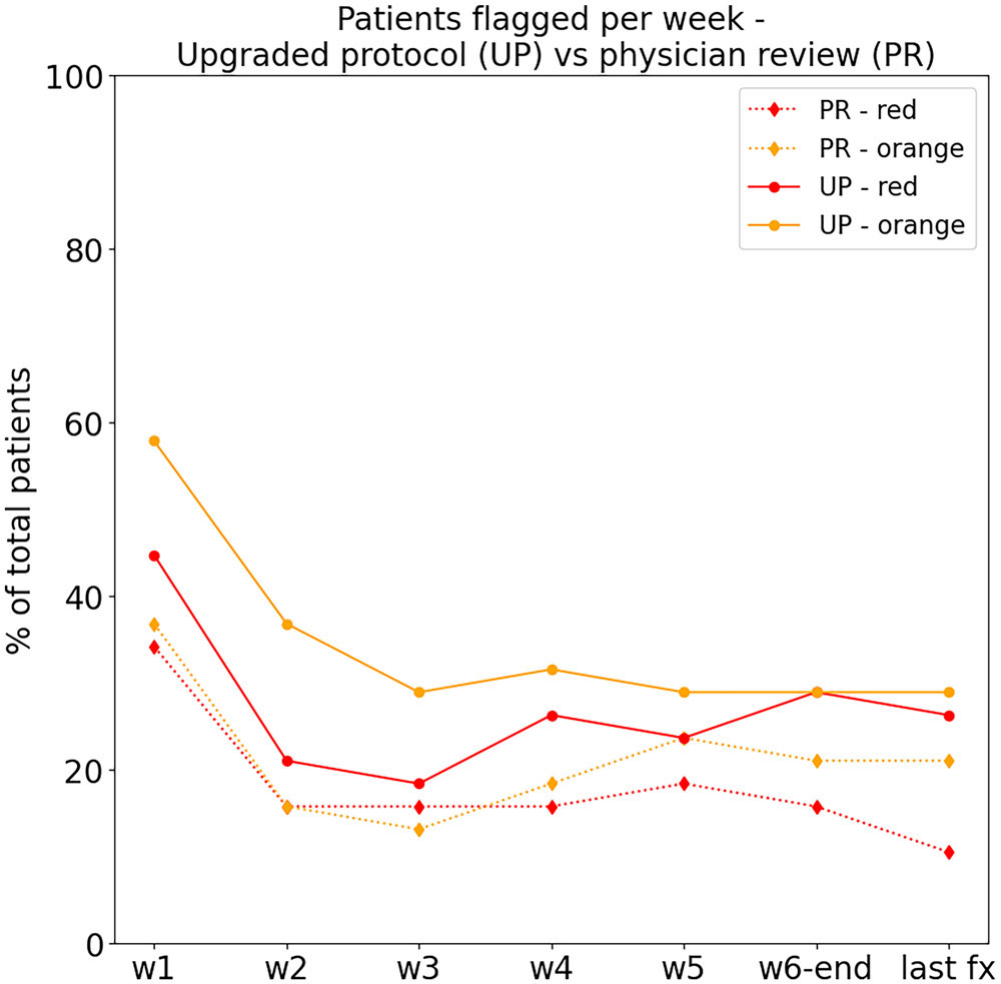
Comparison between the number of flagged patients per week (w1–w6), as well as patients still flagged at the last fraction (last fx), by the physician (PR) and the upgraded protocol (UP).

Regarding the type of organ flagged, 91.65% and 8.35% of red flags corresponded to the PTV and OAR (mandible), respectively; while 100% of orange flags corresponded to the PTV. The number of patients flagged per week by the upgraded protocol in comparison to the physician review is presented in Figure 6. The upgraded protocol overestimated the number of flags, especially for the orange condition at the beginning of the treatment, and for the red condition at the end of the treatment. This overestimation is reflected also in the number of False Positives for each condition (112 for red and 274 for orange).

### 3.4 Application of the upgraded protocol to different definitions of the target volume

**FIGURE 7:**
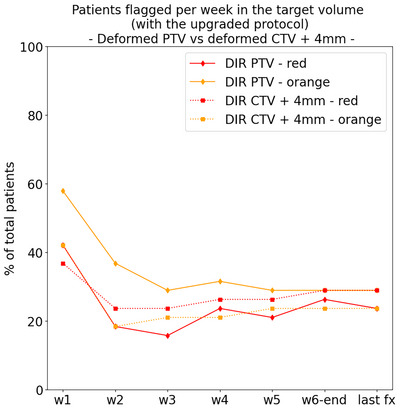
Comparison of the number of patients flagged per week in the target volume with the final (upgraded) protocol: evaluation on the deformed PTV versus the deformed CTV + 4 mm (i.e. the CTV‐to‐PTV margin used for planning).

As explained in Section 2.5, the target volume used during the design of the protocol was a deformed PTV, due to pragmatic reasons regarding the available software and graphical interface. However, a better practice is to use a deformed CTV with a margin expansion. Figure 7 shows the comparison of the number of patients flagged per week in the target volume with the final (upgraded) protocol, using the deformed PTV and the deformed CTV + 4 mm (i.e. the CTV‐to‐PTV margin used for planning). The change of volume led to important differences, particularly for the orange flags in the first weeks, with a difference in flagged patients up to 18% (seven patients) higher for the deformed PTV in w2. The number of patients flagged with orange flags were always higher when looking at the deformed PTV instead of the deformed CTV+4 mm, whereas for red flags the trend was inverted (higher flagging for deformed CTV+4 mm) for all weeks except the first (w1). For the red flags, the differences were less pronounced and always below 8% (three patients).

**FIGURE 8:**
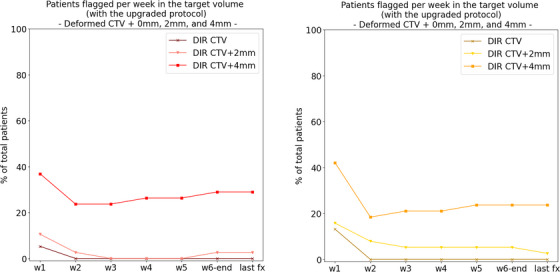
Comparison of the number of patients flagged per week in the target volume with the final (upgraded) protocol: evaluation on the deformed CTV + 0, 2, and 4 mm for red flags (left) and orange flags (right).

Regarding the evaluation of the final protocol for target volumes with reduced margins, Figure 8 shows the results for the number of patients flagged per week in the target volume, using the deformed CTV + 4 mm, a reduced margin of 2 mm, and no margin (the raw CTV). The number of patients flagged per week decreased in median by 26% (10 patients) and 18% (seven patients) for red and orange flags, respectively, when reducing the CTV‐to‐PTV margin from 4 to 2 mm. This resulted in only one patient flagged at the last fraction for both red and orange flags. Decreasing the margin further towards the raw CTV did not influence much the number of flagged patients, with a median reduction of 3% (one patient) for red flags and 5% (two patients) for orange flags.

## 4 DISCUSSION

This manuscript presents and analyses the different steps undertaken to design a clinical protocol for dosimetrically triggered ART. Specifically, this work underlines the complexity of designing general flagging conditions and demonstrates how a theoretical protocol can be adapted, following an iterative process, to match the clinical needs (represented in this case by the physician's answers). This iterative process involved the review of the protocol by a multidisciplinary team and should be performed for every theoretical protocol before envisaging any clinical implementation. During the iterative process, we recommend to first use a retrospective patient cohort and encourage one or several physicians from the team to perform the review process. By doing so, the translation of the physicians’ actions into mathematical rules comes naturally, as we tend to be systematic when performing the same action (flag) multiple times (for every patient, organ and fraction). We believe that the use of retrospective data for this end is a very good practice for the medical doctors and might be safer than performing this exercise directly in a prospective manner. In fact, using retrospective data and analysing the results as a multidisciplinary team might help to have improved, consensual, and more systematic clinical decisions. Eventually, the physician(s) in charge of the review process might experience a “learning/stabilising” curve while attributing the flags for the different organs, fractions, and patients, that is the first flags might be more inconsistent than the last ones, where the physician has learn to recognise patterns and maintain a consistent behaviour. The physician might start by training him/herself with a smaller set of patients, and begin the review process only when he/she can ensure a high consistency in the clinical decisions. This will definitely help to efficiently translate the clinical decisions into mathematical rules, since the more the decisions are inconsistent the more an algorithm will struggle matching the clinician's behaviour.

Please note that, in order not to bias the clinical judgement during this study, the actual adaptation rate on the retrospective database (i.e. back in the time when the patients were treated) was never shown nor taken into account. Indeed, the former clinical decisions do not reflect the current clinical environment anymore, since those patients were treated before the automatic software for ART was acquired. Back in time, the adaptation rate was conditioned by the lack of resources (human, software, hardware) and adaptation only happened in very extreme cases. Today, we can offer to the patients a more precise treatment, and the adaptation rate is not conditioned to the available resources anymore but to the actual deviations between the daily and the planned dose, which is the topic of this study.

In this work, only one physician performed the review process, but ideally, several physicians should be involved to perform the review independently or as a group, thereby building an even more robust protocol, based on the consensual feedback of all involved physicians. In our case, a single loop (iteration) was enough to tune the initial protocol to match the physician's answers to reduce the number of False Negatives and False Positives. The upgraded protocol achieved a sensitivity equal to 0.84 for orange flags and 0.96 for red flags, and a global sensitivity equal to 1 (only one FN), which we considered enough for clinical implementation. But depending on the case, several loops (iterations) can be performed in order to further tune the rules to improve the sensitivity towards the desired minimum value.

A good flagging system is definitely a valuable tool to help the clinical decision, but our results highlight that choosing proper flagging criteria is crucial in order to avoid misusing this tool. On one hand, the analysis of the three difference tolerances for the initial protocol demonstrated that using very conservative criteria entails the risk of flagging almost the totality of the patients (>75% for orange flags with tol = 2.5%), while less conservative levels may instead miss some patients that actually would have benefit from replanning. On the other hand, the initial protocol demonstrated to be too simplistic, since it applied the same rules for all types of volumes. Therefore, we strongly recommend to elaborate dosimetric rules distinguishing between targets, serial, and parallel organs, as specified in the upgraded protocol, and that eventually combine several DVH metrics rather than a single one to trigger a flag.

For simplicity, both the implemented protocol and the physician evaluated each fraction independently, that is the protocol was blind to the number of flags in the previous fractions and the fraction number. One could actually either look at the previous fractions and build flagging criteria taking into account any possible trend, or either apply different flagging criteria depending on the treatment week (i.e. beginning, middle, or end of the treatment). The former might entail some risks, since detecting dosimetric trends is not straightforward. However, the latter may actually help to build a more clinically meaningful protocol, since performing adaptation after a certain fraction may not make sense (e.g. adapting in the very last fractions).

Despite the large number of groups investigating the need of ART for H&N through in‐silico trials or even performing ART clinically,^7^ there are few publications reporting the actual dosimetric criteria and a lack of international consensus or guidelines regarding dosimetrically triggered ART. This results in a variety of heterogeneous results reported in the literature and a lack of reproducibility,^29^ due to the diverse selection criteria used by each research group. The rules implemented in the upgraded protocol can serve as a starting point for other centres wishing to implement dosimetrically triggered ART. However, due to the fact that there were very few organs flagged by the physician during the review process, the OAR criteria presented here should not be taken as solid flagging rules, since they could not be tuned properly with such few samples. In any case, guidelines elaborated by groups of experts (for instance, ICRU, ESTRO or AAPM groups) to establish lower and upper tolerance levels are very needed in order to homogenise the flagging criteria and get rid of the inter‐centre variability.

The system used for daily dose evaluation has a couple of limitations. First, the contours for every fraction were obtained by propagating the contours from the planning CT through deformable image registration (DIR). However, it is well‐known that DIR algorithms may fail in the presence of large deformations of the patient anatomy or tissue creation/deletion. In our case, an experienced physician performed the visual inspection of all contours. But ideally, automatic solutions for quality assurance of DIR should be implemented to avoid this manual and time‐consuming task, which often hampers its clinical implementation. Alternative solutions to get the daily contours use the recent and promising deep learning methods for automatic segmentation.^30^, ^31^, ^32^, ^33^, ^34^, ^35^ Second, MVCT images display poorer contrast than CT scanners and kV‐CBCT scanners, which might influence the quality of contours and the generated DIR field. Moreover, the quality and stability of image quantification for dose calculation has been a matter of concern for the Tomotherapy system, although several upgrades and quality control protocols have considerably mitigated this issue.^36^, ^37^, ^38^, ^39^

The present manuscript focuses on finding a comprehensive methodology to translate the clinical knowledge into mathematical flagging rules to build a clinical ART protocol, rather than tackling the issues related to the dose evaluation and computation. However, a potential limitation of this study is related to the fact that the flagging criteria for the target volume when designing the protocol have been based on a daily PTV volume obtained after deformable registration from the planning PTV. However, as previously mentioned (Sections 2.5 and 3.4), we believe that the choice of volume to define the protocol is irrelevant as long as the physician applies the same rules to every target volume definition. This has been discussed internally and the physician confirmed that she would have applied the same flagging rules, regardless of the use of a deformed_PTV or a deformed_CTV+ margin. Nevertheless, we wanted to evaluate the effect on the number of flagged patients with the final protocol when choosing a different volume, which was presented in Section 3.4. The results showed that the choice of volume is of crucial importance when evaluating the number of flagging patients. On one hand, using a deformed CTV with a 4 mm margin expansion instead of a deformed PTV led to important differences, up to 18% (seven patients) less flagged patients (orange flags). On the other hand, it is well‐known that one of the potentials of daily adaptation is to enable a margin reduction, by removing the contribution of interfractional setup errors from the CTV‐to‐PTV margin. In order to illustrate this, we evaluated the final protocol in a deformed CTV with a 2 mm margin expansion, as well as in the raw deformed CTV (without margin expansion). Reducing the CTV‐to‐PTV margin from 4 to 2 mm led to a significant decrease in the number of flagged patients, with 26% (10 patients) and 18% (seven patients) less flagged patients in median for red and orange flags, respectively. This highlights again the importance of choosing the correct target volume for dose evaluation in ART, and the potential gains of margin reduction.

A few words should be said about the way the final dose is estimated in this study. In the proposed workflow, the final dose was estimated at each fraction assuming that the remaining fractions would deliver an average of the dose per fraction delivered so far (Section 2.1). The results show that this strategy overestimated the deviations at the end of the treatment in the first week (Fractions 1–5). However, it provided a better estimation of the final flagging rate from week 2 onwards. Other alternative approaches could be used to estimate the final dose, such as projecting the last delivered fraction until the end of the treatment or assuming flawless delivery (i.e. the planned dose) for the remaining fractions. Our method might be slightly more robust since it can take into account some trend (overdose/underdose) in the already delivered fractions that could be reproduced in the rest of the treatment.

To finish, some other clinical conditions may influence the physician's decision on the need for adaptation (i.e re‐irradiation or pre‐existing organ dysfunctions). The dosimetrical rules proposed in this manuscript could be combined with machine learning and artificial intelligence methods,^40^ to design more sophisticated workflows that are able to grab patient‐specific information and mimic the physician decision process on whether to replan or not in a more reliable way.

## 5 CONCLUSION

The number of decision support systems for adaptive radiotherapy, some of them embedded in commercial treatment planning systems, has increased in the last years. These software provide excellent tools to analyse daily images and doses, but leave (if even allowed) the criteria to trigger plan adaptation to each centre appreciation. This work presents a three‐steps iterative process, involving a multidisciplinary team, where an initially designed theoretical protocol is applied to a retrospective database and tuned to match the clinical needs. Our results demonstrate the feasibility of using simple mathematical rules to mimic the physician's decisions regarding ART, reaching a very high sensitivity. We believe this work could help those centres willing to implement ART in the process of designing specific clinical protocols, and contribute to the standardisation of the clinical practice of ART. This would increase ART efficiency, especially when facing time or teams shortage. We recommend performing this iterative benchmark process for every ART protocol, before envisaging any clinical implementation.

## AUTHOR CONTRIBUTIONS

E.S. and X.G. devised the project, the main conceptual ideas and proof outline. A.B.M. and D.D. worked on the technical details, and performed the numerical calculations for the experiments (database processing, daily dose recomputation and analysis of results). G.V.O. performed the flagging exercise on the whole database, with the support of S.T.R. G.V.O. and S.T.R. also supported A.B.M. in the database review and analysis of results. A.B.M. was in charge of writing the manuscript but the rest of the authors contributed with revision and valuable feedback regarding the content.

## CONFLICT OF INTEREST STATEMENT

The author declares no conflicts of interest.
